# Idebenone Protects against Retinal Damage and Loss of Vision in a Mouse Model of Leber’s Hereditary Optic Neuropathy

**DOI:** 10.1371/journal.pone.0045182

**Published:** 2012-09-18

**Authors:** Fabrice D. Heitz, Michael Erb, Corinne Anklin, Dimitri Robay, Vincent Pernet, Nuri Gueven

**Affiliations:** 1 Santhera Pharmaceuticals, Liestal, Switzerland; 2 Brain Research Institute, Medical Faculty of the University of Zürich, Zürich, Switzerland; 3 School of Pharmacy, University of Tasmania, Hobart, Australia; Charité University Medicine Berlin, Germany

## Abstract

Leber’s hereditary optic neuropathy (LHON) is an inherited disease caused by mutations in complex I of the mitochondrial respiratory chain. The disease is characterized by loss of central vision due to retinal ganglion cell (RGC) dysfunction and optic nerve atrophy. Despite progress towards a better understanding of the disease, no therapeutic treatment is currently approved for this devastating disease. Idebenone, a short-chain benzoquinone, has shown promising evidence of efficacy in protecting vision loss and in accelerating recovery of visual acuity in patients with LHON. It was therefore of interest to study suitable LHON models *in vitro* and *in vivo* to identify anatomical correlates for this protective activity. At nanomolar concentrations, idebenone protected the rodent RGC cell line RGC-5 against complex I dysfunction *in vitro.* Consistent with the reported dosing and observed effects in LHON patients, we describe that in mice, idebenone penetrated into the eye at concentrations equivalent to those which protected RGC-5 cells from complex I dysfunction *in vitro*. Consequently, we next investigated the protective effect of idebenone in a mouse model of LHON, whereby mitochondrial complex I dysfunction was caused by exposure to rotenone. In this model, idebenone protected against the loss of retinal ganglion cells, reduction in retinal thickness and gliosis. Furthermore, consistent with this protection of retinal integrity, idebenone restored the functional loss of vision in this disease model. These results support the pharmacological activity of idebenone and indicate that idebenone holds potential as an effective treatment for vision loss in LHON patients.

## Introduction

Leber’s hereditary optic neuropathy (LHON) is a rare inherited mitochondrial disorder with an estimated prevalence in Europe of 1∶45000 [1] characterized by rapid loss of visual acuity and color contrast sensitivity leading to blindness [2], [3]. While generally this blindness persists lifelong, there is a limited rate of spontaneous recovery. LHON predominantly affects young adult males in all ethnic groups, with a peak of onset between the ages of 15–30 years. LHON starts with an acute onset of vision loss, first in one eye, followed by second eye involvement, typically within weeks to a few months. In most cases the vision loss is permanent, although a minority of patients shows spontaneous recovery of visual acuity [4], [5]. LHON patients show swelling of the retinal nerve fiber layer with pseudoedema of the optic disc and a circumpapillary telangiectatic microangiopathy during the acute stage of the pathology. Retinal ganglion cell (RGC) bodies and their projecting axons forming the retinal nerve fiber layer (RNFL) gradually degenerate during the chronic phase with atrophy of the temporal optic nerve or diffuse optic atrophy [6], preferentially affecting the small-caliber fibers of the papillomacular bundle (PMB) at the center of the retina [7].

LHON is caused by well characterized mitochondrial DNA (mtDNA) mutations, among which three so called primary mtDNA mutations account for more than 95% of all LHON cases: 11778G>A (ND4 subunit) [8], 14484T>C (ND6 subunit) [9], and 3460G>A (ND1 subunit) [10]. The 11778G>A mutation generally is the most abundant, although there is considerable variation in the relative frequency of the three primary LHON mutations worldwide. The degree of severity of the disease largely depends on the individual mutation, with the 14484T>C demonstrating a milder phenotype with a higher frequency of spontaneous recovery. All the mutations affect genes encoding mitochondrial complex I subunits (NADH dehydrogenase), which is part of this large multi-enzyme assembly that generates ATP as a cellular energy source through electron transfer and oxidative phosphorylation (OXPHOS). Therefore not surprising, defects in mitochondrial energy production have been reported for cells harboring the LHON mutations [11]. Experimental evidence connects complex I dysfunction to decreased ATP synthesis, elevated levels of oxidative stress [12], [13] and impaired glutamate transport [14] leading to RGC dysfunction and ultimately to apoptotic cell death [15]. Recently developed murine animal models of LHON that mimic essential aspects of pathology of LHON pathology can be used to test possible treatment strategies for LHON. One of these mouse models relies on intravitreal injection of the complex I inhibitor rotenone which causes loss of RGCs accompanied by reduced retinal nerve fiber layer thickness, thereby mimicking the cellular pathology of LHON patients [16], [17], [18], [19], [20]. Consistent with this, complex I inhibition by rotenone leads to ROS-mediated toxicity and apoptotic cell death in vitro, which are also observed in LHON [21].

Idebenone, a short-chain benzoquinone, is a potent antioxidant and inhibitor of lipid peroxidation [22], [23], [24] and thus is able to protect cell membranes and mitochondria from oxidative damage. Idebenone also interacts with the mitochondrial electron transport chain and modulates mitochondrial electron flux [25]. Specifically, it has been suggested that idebenone is effective in by-passing complex I to maintain cellular energy production. This bypass of complex I was first suggested by [26] and recently described in detail by [27] who demonstrated that idebenone is efficiently reduced in the cytoplasm by NAD(P)H quinone oxidoreductase 1 (NQO1) to the hydroquinone form. Under conditions of impaired complex I function, this reduced form of idebenone is able to transport electron equivalents from the cytoplasm to the mitochondria, where they are directly fed into the respiratory chain at the level of complex III. This mechanism of by-passing a defective complex I, which effectively restores cellular ATP levels [27], [28], together with its antioxidant function, provides a strong rationale for the use of idebenone in LHON.

Here we describe the pharmacological effects of idebenone in an RGC cell line and a well-established mouse model of LHON and demonstrate the protective effect of idebenone on retinal toxicity and visual impairment induced by complex I dysfunction.

## Materials and Methods

### Test Compound, Formulation and Chemicals

For mouse dietary treatment, idebenone (2-(10-Hydroxy-decyl)-5,6-dimethoxy-3-methyl- [1], [4]benzoquinone) was mixed with 0.5% carboxymethyl cellulose (CMC), food powder (made from autoclaved food pellets) and sucrose (at a final concentration of 4%). Individual portions dispersed in small dishes and containing a daily dose of idebenone from 20 to 2000 mg/kg body weight were administered to single caged animals. A mixture of 0.5% CMC, food powder and sucrose (4%) was used as vehicle. In addition to the food mixture, idebenone suspension was prepared for p.o. (gavage) application by mixing idebenone with 0.5% CMC. Finally, a volume to result in a 60 mg/kg dose was applied by gavage. For cell culture experiments, idebenone was dissolved at 1 mM (stock solution) in 100% DMSO (Acros Organics, Belgium) and the final concentration of DMSO did not exceed 0.1% in the culture medium. For mouse intravitreal injection, rotenone was dissolved at 5 mM in 100% DMSO. DMSO was used as a control for the sham injection. All other chemical reagents and cell culture media and solutions, if not otherwise stated, were purchased from Sigma (Sigma-Aldrich, Buchs, Switzerland) and PAA Laboratories GmbH (Pasching, Austria).

**Figure 1:**
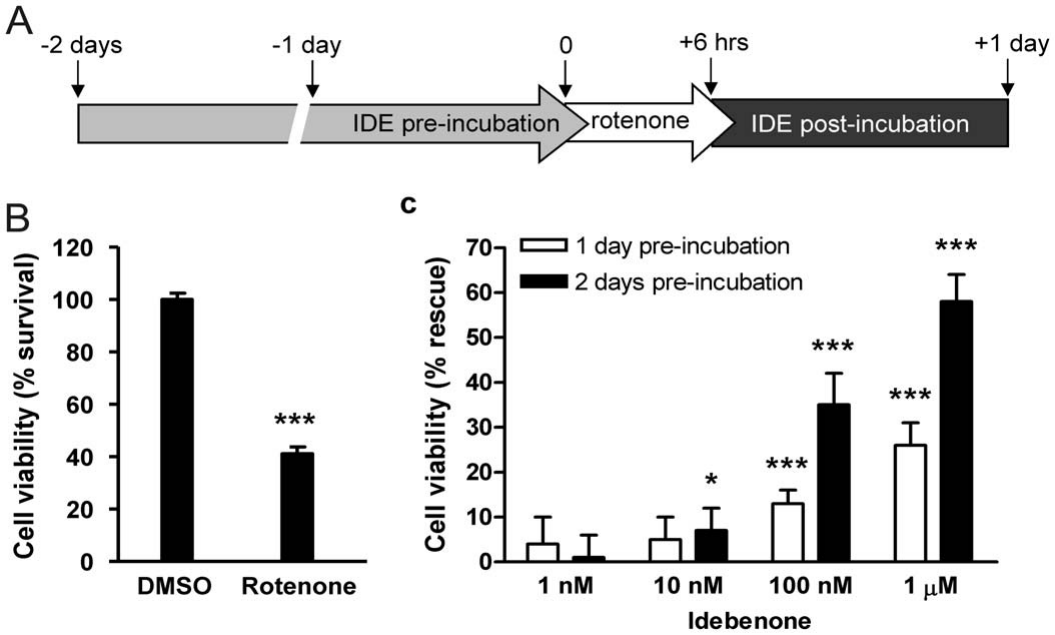
Protective effect of idebenone against complex I inhibition in RGC-5 cells *in vitro*. (A) Scheme of the experimental design. RGC-5 cells were pre-incubated with idebenone or vehicle (DMSO) for 1 or 2 days before the complex I inhibitor rotenone was applied to the cells for 6 hours. Cell viability was then measured after 1 day post-incubation with idebenone or vehicle. (B) Treatment with rotenone caused a 59% reduction in cell viability in vehicle-treated RGC-5 cells. (C) Idebenone pre-treatment for 1 (white bars) and 2 days (black bars) significantly rescued cell viability (expressed as % rescue). Data are expressed as mean ± SD (n = 6 wells per group). Statistical significance relative to vehicle group: p≤0.05 (*), p≤0.001 (***).

### Cell Culture

RGC-5 cells were cultured under standard conditions (37°C, 5% CO_2_, 90% humidity) in DMEM (1 g/l glucose; 10% foetal bovine serum (FBS), Penicillin-Streptomycin-Glutamine (PSG)). For experiments, 3×10^3^ RGC-5 cells per well were seeded in DMEM (1 g/l glucose; 2% FBS, PSG) into BD Falcon™ 96-well plates. RGC-5 cells were constantly exposed to either idebenone or vehicle from one day after seeding (pre-incubation), together with rotenone (co-incubation) or after the rotenone stress (post-incubation). For the 1 day post-incubation period, cells were cultured in Hanks’ BSS (1 g/l glucose, PSG, idebenone or vehicle).

**Figure 2:**
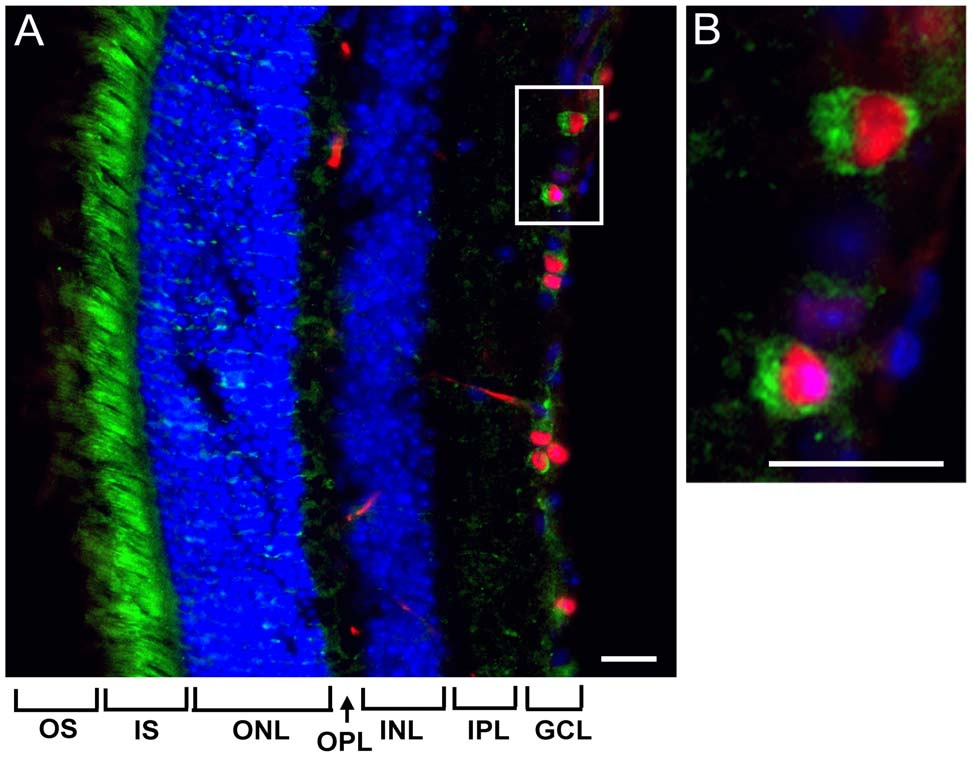
NQO1 expression in RGCs and the inner segment of photoreceptors. Distribution of NQO1 in the mouse retina co-stained with rabbit anti-NQO1 and mouse monoclonal anti-Brn3a primary antibodies. Anti-rabbit Alexa 488-conjugated and anti-mouse Alexa 495-conjugated secondary antibodies were used for fluorescent visualization of NQO1 (green) and RGCs (red) in the retina. The white rectangle in (A) depicts the region of interest shown at high magnification in (B). The different retinal cell layers are shown by nuclear staining using DAPI (blue). Scale bars represent 30 µm. (OS: outer segment, IS: inner segment, ONL: outer nuclear layer, OPL: outer plexiform layer, INL: inner nuclear layer, IPL: inner plexiform layer, GCL: ganglion cell layer (incl. nerve fiber layer).

### Cell Viability Analysis

As a measure of cell viability, the cellular ATP content was quantified by luminescence from the ATP-dependent enzymatic oxidation of luciferin by luciferase and using a multimode plate reader (Tecan M1000, Tecan iControl 1.6 software; Tecan, Groedig, Austria). Briefly, RGC-5 cells were washed in PBS and lysed in 100 µl lysis solution (4 mM EDTA, 0.2% Triton X-100) for five minutes at room temperature. In 96-well plates, 100 µl of ATP measurement buffer (25 mM HEPES pH 7.25, 300 µM D-luciferin, 5 µg/ml firefly luciferase, 75 µM DTT, 6.25 mM MgCl_2_, 625 µM EDTA and 1 mg/ml BSA) was combined with 10 µl cell lysate to start the reaction. Idebenone-mediated rescue in cell viability was defined as the percentage of idebenone-mediated increase in cell viability (based on ATP content) relative to the rotenone-induced reduction in cell viability (based on ATP content).

**Figure 3:**
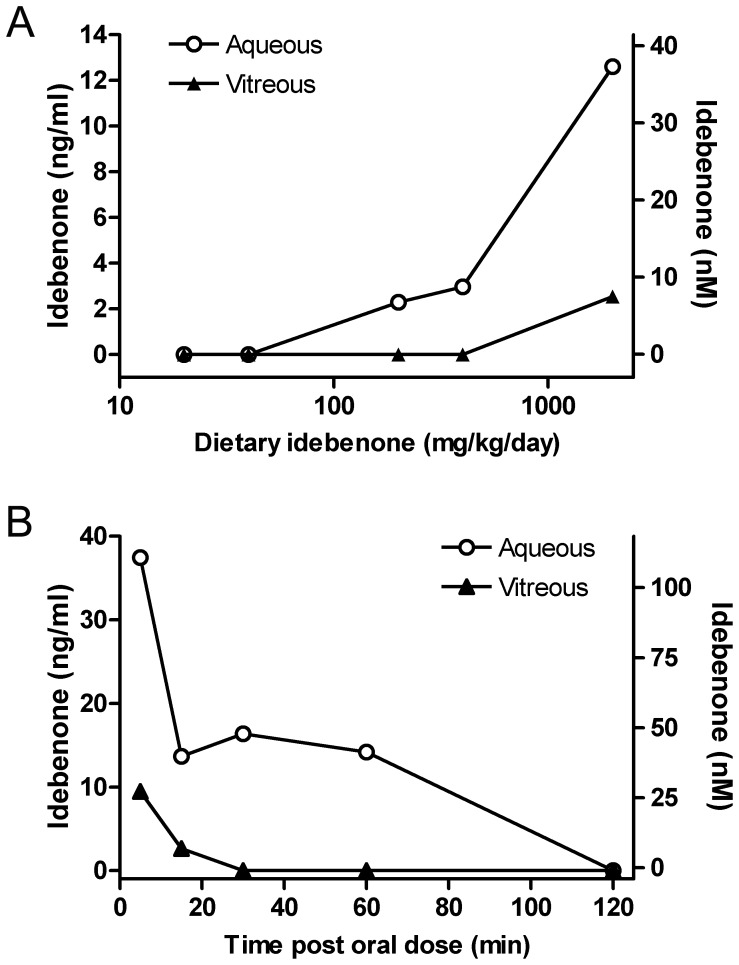
Pharmacokinetic analysis of idebenone in eye fluids. Concentrations of idebenone in aqueous humor (open circle) and vitreous humor (filled triangle) following once daily administration of idebenone in the diet for 21 days (A) and following single oral administration of idebenone at 60 mg/kg (B) were determined. Concentrations of idebenone in the eye fluids are expressed as ng/ml (left y axis) and nM (right y axis). For (A), sampling time was more than 8h after last dose of idebenone, the values therefore represent trough levels.

### Animals

Male wild-type C57BL/6J mice (7 weeks old, 25 g body weight) obtained from Janvier (France) were single-housed in Makrolon type II cages under a regular 12-hours light-dark cycle (8 am to 8 pm) with free access to food and water. All procedures were performed in accordance with the Swiss regulation on the use and care of animals and under the required license approved from the Kantonales Veterinäramt Basel-Land (permission Nr. 415).

**Figure 4:**
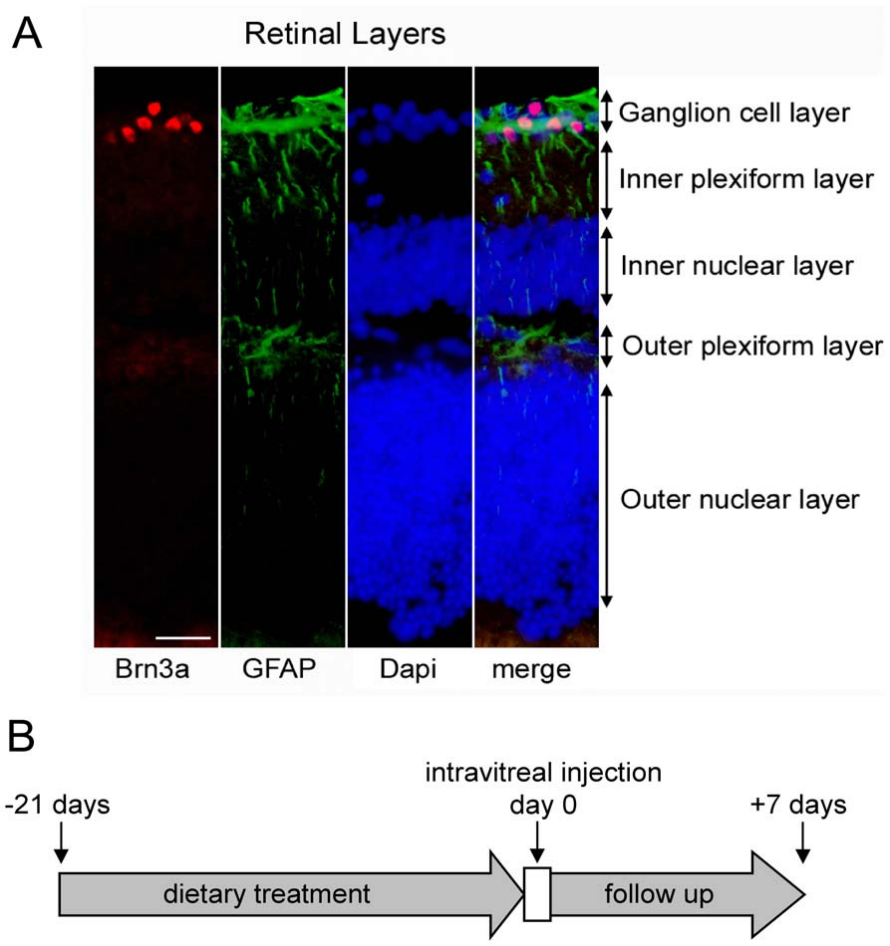
Experimental approach to study the protective effect of idebenone in a LHON mouse model. (A) Image of a normal retinal section showing RGCs (red) and Müller glial cell projections (green) using anti-Brn3a and anti-GFAP primary antibodies respectively. The different retinal cell layers are shown by nuclear staining using DAPI (blue). (Scale bar = 25 µm). (B) To investigate the efficacy of idebenone in protecting against complex I deficiency in the retina *in vivo*, we performed intravitreal injection of rotenone (15 mM) in mice pre-treated for 21 days with idebenone or vehicle in diet. Retinal ganglion cell number, retinal thickness and reactive gliosis were evaluated based on histological analysis of retinal sections 7 days after rotenone challenge.

**Figure 5:**
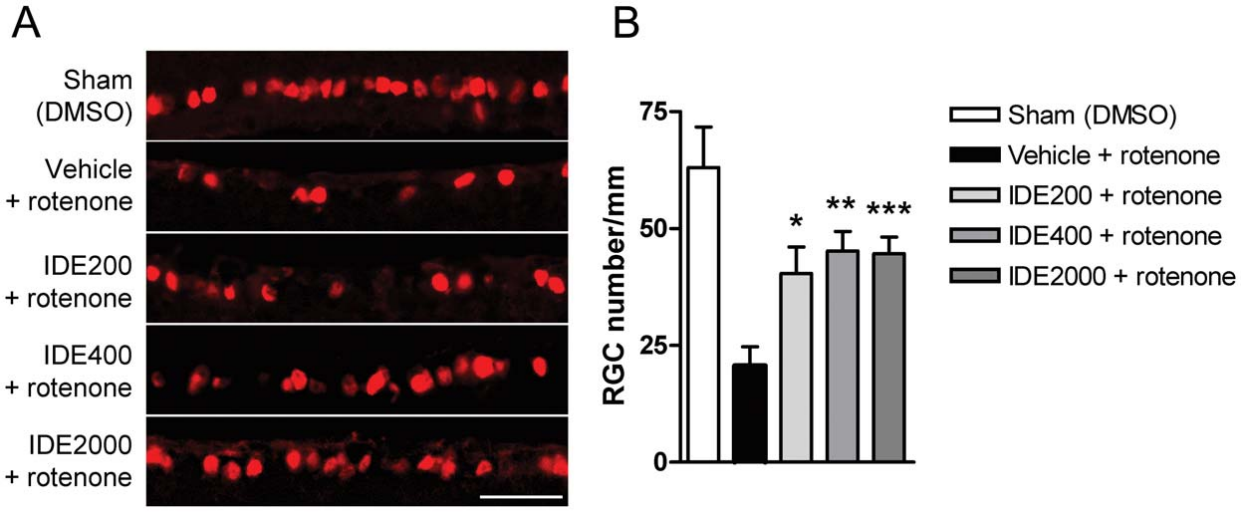
Idebenone prevents rotenone-induced RGC death. Analysis of RGCs in the mouse retina 7 days after intravitreal injection of rotenone or DMSO (Sham). Mice were treated with dietary idebenone (IDE 200, 400 and 2000 mg/kg body weight) or vehicle. (A) Images of retinal slices stained with anti-Brn3a primary antibody to visualize RGCs. Scale bar = 25 µm. (B) Quantification of RGCs (RGC number/mm) following idebenone treatment and rotenone injection (n = 6 to 10 per group). Data expressed as mean ± SEM.

**Figure 6:**
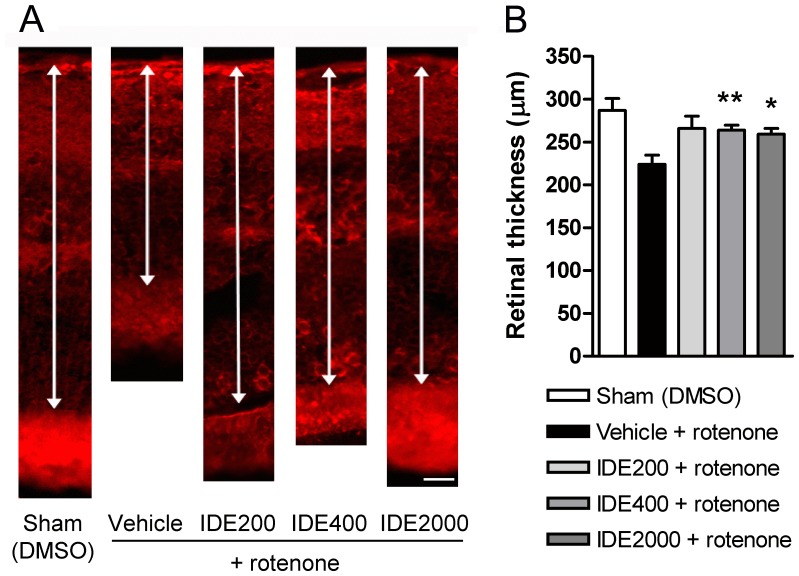
Idebenone protects against rotenone-induced decrease in retinal thickness. For analysis of retinal thickness 7 days after intravitreal injection of rotenone or DMSO (Sham), mice were treated with dietary idebenone (IDE 200, 400 and 2000 mg/kg body weight) or vehicle. (A) Images of retinal slices stained with anti-beta 3 tubulin primary antibodies to visualize inner and outer retinal layers. The arrows indicate retinal thickness measured from the pigment epithelium to the nerve fiber layer. Scale bar = 30 µm. (B) Quantification of total retinal thickness (µm) following idebenone treatment and rotenone injection (n = 6 to 10 per group). Data are expressed as mean ± SEM.

**Table 1:** Dose-effect of idebenone treatment on multiple endpoints in an *in vivo* mouse model for LHON.

Treatment	RGC number	Retinal thickness	Reactive gliosis
**Sham (DMSO)**	100.0±13.7 (n = 10)	100.0±4.9 (n = 8)	100.0±9.7 (n = 7)
**Vehicle + rotenone**	32.8±6.4 (n = 6)	78.1±3.8 (n = 6)	178.1±21.6 (n = 3)
**IDE200 + rotenone**	64.0±9.1* (n = 10)	92.7±4.9 (n = 10)	124.5±7.4* (n = 7)
**IDE400 + rotenone**	71.7±6.7** (n = 8)	91.9±2.0** (n = 8)	125.0±9.1* (n = 6)
**IDE2000 + rotenone**	70.8±5.6*** (n = 10)	90.3±2.3* (n = 9)	129.9±7.3* (n = 7)

Histological data are expressed as % of Sham (DMSO) controls ± SEM. Numbers of retinal ganglion cells (RGC number) is based on RGCs/mm 7 days post-injection applicable to all groups; retinal thickness (µm) 7 days post-injection; gliosis is based on relative fluorescence units (RFU) of GFAP-specific immunostaining 7 days post-injection. Sham (DMSO): sham-injected with DMSO; vehicle: vehicle treated; rotenone: rotenone-injected; IDE200: idebenone treated (200 mg/kg); IDE400: idebenone treated (400 mg/kg); IDE2000: idebenone treated (2000 mg/kg). Statistical significance relative to vehicle + rotenone group: p≤0.05 (*), p≤0.01 (**), p≤0.001 (***); based on raw numbers; n = number of retinas per group.

**Figure 7:**
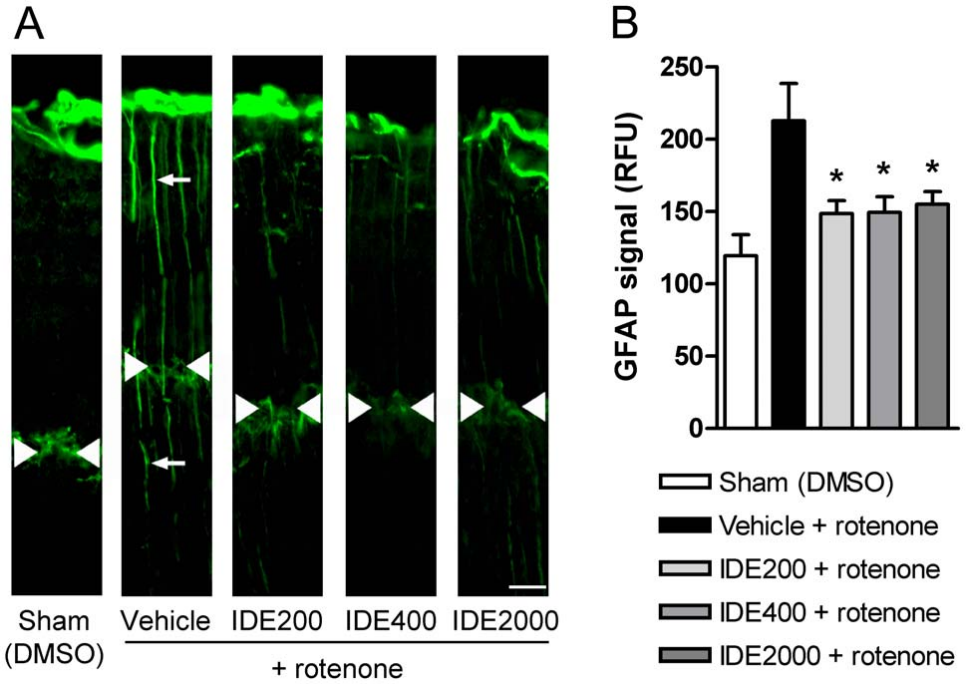
Idebenone protects against rotenone-induced gliosis. For analysis of gliosis 7 days after intravitreal injection of rotenone or DMSO (Sham), mice were treated with dietary idebenone (IDE 200, 400 and 2000 mg/kg body weight) or vehicle. (A) Images of retinal slices stained with anti-GFAP primary antibody to visualize Müller glial cell projections (white arrows). White arrowheads indicate outer plexiform layer and also illustrate rotenone induced reduction in retinal thickness (i.e. distance between white arrow heads and ganglion cell layer at the top of the images). Scale bar = 15 µm. (B) Quantification of gliosis (GFAP signal based on relative fluorescence units, RFU) following idebenone treatment and rotenone injection (n = 3 to 7 per group). Data are expressed as mean ± SEM.

### Pharmacokinetics

Mice were treated daily with dietary doses of idebenone or vehicle for 21 days or were treated orally by gavage with a single 60 mg/kg dose of idebenone. Mice were sacrificed and biological fluids were sampled 17 to 20 hours following dietary treatment and at time intervals from 5 min to 6 hours after gavage dosing. Briefly, mice were anesthetized by 5% isofluran inhalation in an induction chamber and sacrificed by cervical dislocation followed by decapitation. Blood was collected from the head into EDTA-containing tubes for plasma preparation. Individual plasma samples (Table S1) or pooled plasma samples from 4 individual mice (Table S2, S3) were analyzed. Both eyes were dissected out for the collection of aqueous and vitreous humor. Due to low individual collection volumes, aqueous humor and vitreous humors samples were pooled from 20 and 10 individual mice, respectively. Samples were transferred into 1.5 ml microcentrifuge tubes and stored frozen at -80°C until analyzed. Idebenone content was quantified by Inovalab AG Bioanalytics (Switzerland) using validated LC-MS/MS methods for plasma, aqueous humor and vitreous humor. The lower limit of detection of idebenone was determined as 2 ng/ml. Concentrations below this limit of detection were set as zero for the concentration summary statistics. Pharmacokinetic data are presented as descriptive statistics.

**Figure 8:**
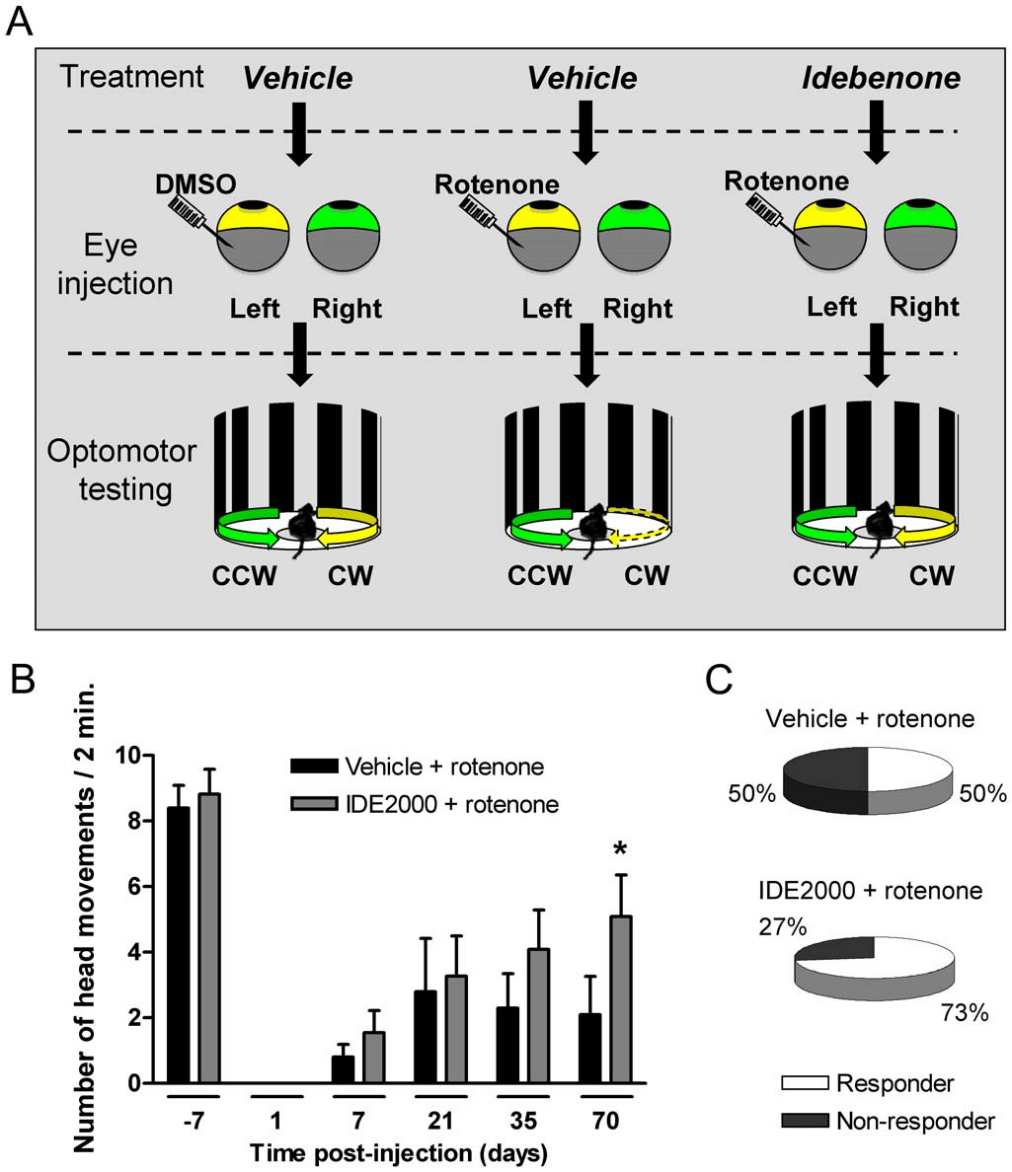
Idebenone time-dependently restores rotenone-induced loss of vision. Visual acuity was evaluated by counting the number of head movements at a velocity of 3 rpm 7 days prior injection (day -7) and 1 to 70 days after injection (day 1, 7, 21, 35, 70) of rotenone (5 mM). Mice were treated with dietary idebenone 2000 mg/kg or vehicle for the time of the experiment. (A) Schematic representation of the experimental setup. After vehicle (DMSO) injection, head movements in both directions of drum rotation (clockwise: CW; counterclockwise CCW) remain intact. Injection of rotenone into the left eye however, affects only the CW responses (dashed arrow) whereas CCW responses remain unaffected. (B) Quantification of clockwise (CW) head movement (number of head movements/2 min) following vehicle treatment and rotenone injection (vehicle + rotenone, n = 10 animals), and idebenone 2000 mg/kg treatment and rotenone injection (IDE 2000+ rotenone, n = 11 animals). Data are expressed as mean ± SEM. Statistical significance relative to vehicle + rotenone group: p≤0.05 (*); (C) Percentage of mice showing clockwise (CW) head movements for each treatment group 70 days after injection. Responder: group of mice showing head movements; Non-responder: group of mice without head movements.

### Intravitreal Injection

Intravitreal injection of rotenone (5 and 15 mM in DMSO) or DMSO (vehicle) was performed as previously described [16], [17]. Briefly, mice were anesthetized by inhalation with 1.5% to 2% isoflurane before 1 µl of rotenone was injected into the vitreous chamber of the left eye using an intraocular injection system (IO-KIT, World Precision Instruments, USA) adapted with a 35-gauge needle, and coupled to a 10-µl syringe (NanoFil, World Precision Instruments, USA). The needle tip was inserted into the superior hemisphere of the left eye, at the level of the pars plana, at a 45° angle through the sclera into the vitreous body. This route of administration avoids retinal detachment or injury to eye structures, including the lens and the iris. The injection was performed using a syringe infusion pump (VIT-FIT, Lambda, USA) over a period of 2 min. The needle was kept in place for an additional 3 min, after which it was gently removed. Surgical glue (Indermill, Tyco Health Care, USA) was used to seal the site of injection and Neosporin eye drops (Neosporin, GlaxoSmithKline AG, Switzerland) were applied to prevent any infection.

### Immunohistochemistry

Mice were anesthetized by i.p. injection of ketamine/xylazine (2/1, 200 µl) 7 days after rotenone injection and transcardially perfused with 4% PFA in PBS 0.1 M (pH 7.4). The left eyes (injected) and the right eyes (uninjected controls) were rapidly removed and the corneas were eliminated at limbus level; vitreous humor and crystalline lens were discarded. Tissue samples were labeled with random numbers by a person not involved in the study to ensure blinding during the analyses. This person kept the ‘treatment allocation’ list to himself and persons involved in tissue processing and data analysis were blind to the treatment allocation at all times. The samples thus obtained were post-fixed with 4% PFA in PBS 0.1 M (pH 7.4) overnight at 4°C. Samples were soaked in a PBS 0.1 M, 30% sucrose solution for 4 days at 4°C, embedded in freezing resin (OCT compound, Tissue-Tek), cryostat-cut in longitudinal sections (14-µm thick), placed on glass slides and stored at -20°C until stained. Sections were blocked and permeabilized in 0.1 M PBS, 0.3% Triton X-100 (Sigma) and 5% heat-inactivated horse serum (HS; Sigma) for 1 h at room temperature. Slices were then incubated overnight at 4°C with primary antibodies in 0.1 M PBS, 0.3% Triton X-100 and 5% HS. The following primary antibodies were used: mouse monoclonal and rabbit anti-Brn3a (1∶100; Millipore, Mab1585, [29]) to stain for RGCs, rabbit anti-GFAP (1∶500; Dako, Z0334) to stain for gliosis, mouse anti-beta 3 tubulin (1∶500; Promega, G7121) to stain for all retinal layers to allow measurement of retinal thickness, and rabbit anti-NAD(P)H-quinone oxidoreductase 1 (NQO1, 1∶100; Sigma, HPA007308) to stain for NQO1 expression in the retina. Slices were then washed three times in 0.1 M PBS and incubated 1 h at room temperature with goat anti-mouse Alexa 594, donkey anti-mouse 488, and donkey anti-rabbit Alexa 488 fluorescence-conjugated secondary antibodies (1∶2000; Invitrogen) in 0.1 M PBS, 0.3% Triton X-100 and 5% HS. DAPI (Invitrogen) was used for the staining of cell nuclei. After washing three times in 0.1 M PBS, slices were mounted using FluorSave reagent (Calbiochem) and stored in the dark at 4°C. Images were acquired with a fluorescent microscope (Olympus) and analyzed using Image J software. Immunostaining analysis of the retinal sections was achieved on the contralateral side of the injection (inferior hemisphere of the eye). For RGC numbers, Brn3a-positive RGCs were counted and presented as number of RGCs per millimeter in the region of interest. Retinal thickness was determined by tubulin staining. Glutamine synthetase (GS) expression and the extent of gliosis was assessed by quantifying specific fluorescence signals within three regions of interest per retinal slice (background fluorescence was subtracted and signals from 3 regions of interest were averaged for each retinal slice). Quantitative analysis was performed on a minimum of two slices per individual retina for each parameter and the mean value was recorded. Image analysis of retinal slices was blinded to ensure that the person performing the analysis was not aware of the treatment received by the animal. After all images were analyzed and quantitative data were recorded, the study was unblinded and the treatment allocation list was matched to the data.

### Optomotor Testing

The optomotor response was measured as previously described [30]. Mice were placed on a central platform (10 cm diameter) surrounded by a motorized drum (29.0 cm diameter) with black and white vertical stripes (1.0 cm thickness) that could rotate both clock- and counterclock-wise at different velocities (which was adjusted in the first experiment to evoke the optimal optokinetic response) under constant light levels (Video S1, S2). This test is particularly suitable for assessing visual acuity under these experimental conditions because the left eye is entirely responsible for controlling the optomotor reflex (head movements) when the drum is rotating clockwise, whereas the reflex is controlled exclusively by the right eye for the counterclockwise rotation. Therefore, the visual acuity of the left (injected) and of the right (non-injected) eye was tested by clock- and counterclock-wise rotation of the drum. Mice were allowed to habituate for 5 min to the experimental setup before the drum was rotated alternately clockwise and counterclockwise, for 2 min in each direction with an interval of 30 s between the two rotations. Movements were digitally recorded using a video camera mounted above the apparatus and the number of head movements was subsequently manually scored. Head movements were scored only when the angular speed of the head turn corresponded to that of the drum rotation. Each mouse was tested at weekly intervals throughout the experiment and the apparatus was cleaned between mice.

Behavioral analysis of the optomotor response was achieved for 1) visual recovery by counting the number of clockwise head movements per 2 min session prior to rotenone injection and at different time intervals following rotenone injection, 2) number of mice showing clockwise head movements at different time intervals following injection and expressed as the percentage of responder mice, and 3) visual acuity of the non-injected eye used as a control by counting the number of counterclockwise head movements per 2 min session prior injection and at different time intervals following injection. The overall experimental process was blinded at each step to ensure that the persons involved in optomotor testing, counting optomotor responses and analyzing the data were not aware of the treatment given to the animals. At the end of the analysis, the study was unblinded and the treatment allocation was matched to the data.

### Exclusion Criteria

To correct for possible deviations in the procedure that could lead to erroneous conclusions, immunolabeled sections were evaluated for staining quality. Specifically, samples were excluded from the quantitative analysis in case of weak or unspecific fluorescence signal defined as relative fluorescence units (RFU) ≤20% of standard values or ≤120% of the background fluorescence. In addition, functional quantification of visual acuity (see *Optomotor testing*) was used to control for successful intraocular rotenone injection. In response to the injection of rotenone, a total loss of visual acuity was generally observed within 24h in the optomotor test system. Consequently, animals not showing complete loss of optomotor response at 24h post rotenone injection were excluded from histological and behavioral analyses.

### Statistical Analysis

Data are presented as mean ± SD or mean ± SEM as indicated in the figure legends. Paired Student’s t-test and analysis of variance (ANOVA) was used to compare non-normalized data. Statistical significance was set at p≤0.05(*), p≤0.01(**) and p≤0.001(***).

## Results

### Idebenone Increases Survival of Complex I Deficient RGC-5 Cells

To assess a cytoprotective effect of idebenone, we recapitulated the situation of mutated mitochondrial complex I in LHON RGC cells by exposing the retinal ganglion cell line RGC-5 [31] with the mitochondrial complex I inhibitor rotenone [21]. RGC-5 cells were continuously incubated with 1, 10, 100 nM or 1 µM idebenone for 1 or 2 days prior to a 6 hour exposure to rotenone (1 µM). This toxic stimulus was followed by a 24h post-incubation time with or without idebenone before RGC-5 cell viability was quantified by analyzing cellular ATP content (Fig. 1A). While rotenone-mediated complex I inhibition strongly reduced the viability of RGC-5 cells by 59% (Fig. 1B), idebenone treatment dose-dependently increased cell viability (Fig. 1C). Significant protection by idebenone was already seen at concentrations of ≥10 nM. The protective effect was dependent on the duration of idebenone pre-incubation, since pre-incubation with 1 µM idebenone for 1 day (white bars) and 2 days (black bars) increased RGC-5 cell viability by 26% and 58%, respectively. In summary, these results demonstrate that concentrations of idebenone above 10 nM are cytoprotective against complex I inhibition in a RGC cell line *in vitro*.

### NQO1 Expression in the Mouse Retina

We previously reported that next to its antioxidant activity, one important function of idebenone is to bypass a dysfunctional complex I. This mechanism involves an alternate electron flux directly to complex III and can restore cellular ATP levels [27] and largely relies on the presence of NQO1. We thus examined whether significant levels of NQO1 were expressed in the murine retina and specifically in the RGC cells which are most affected in LHON. When untreated mouse retina sections were stained for NQO1 expression, strong immunoreactivity was found predominantly in the RGC cell layer and the photoreceptor layer (Fig. 2A–B). As expected NQO1 staining in RGC cells was located to the cytoplasm (Fig. 2B). At the same time, only low level immunoreactivity for NQO1 was observed in the other retinal layers (Fig. 2A). The presence of strong NQO1-immunoreactivity in RGCs is in agreement with the proposed mode of action whereby idebenone bypasses a deficient complex I.

### Idebenone Penetrates into the Eye Fluids after Oral Administration in the Mouse

Since nanomolar concentrations of idebenone protect against complex I dysfunction and cell death in an RGC-like cell line *in vitro*, we next assessed whether such concentrations of idebenone could be achieved in the mouse eye following oral administration of idebenone. Following treatment with idebenone given in the diet over a period of three weeks or after a single oral administration (gavage) in the adult mouse, the concentrations of idebenone in the aqueous and vitreous humor and plasma were measured by liquid chromatography coupled to tandem mass spectrometry (LC-MS/MS). After administration of idebenone through the diet of male mice from 20 to 2000 mg/kg of body weight/day, concentrations of idebenone in aqueous and vitreous humor increased approximately dose proportionally, similar to the increase observed in the plasma (Fig. 3A and Table S1). The concentration ratios of aqueous humor/plasma and vitreous humor/plasma appeared to be independent of the dose, consistent with an absence of accumulation of idebenone in the eye (data not shown). Concentrations of idebenone in aqueous humor were approximately 2 to 7-fold higher than that in vitreous humor. Importantly, intra-ocular exposure to idebenone reached peak concentrations of 2.3 ng/ml (7 nM), 3.0 ng/ml (9 nM), and 12.6 ng/ml (37 nM) in the aqueous humor at the oral doses of 200, 400, and 2000 mg/kg of body weight/day, respectively (Fig. 3A and Table S1) which are within the range shown to be effective in protecting RGC-5 cells from rotenone-induced cell death *in vitro* (i.e. 10 nM and above, Fig. 1B). We next determined the concentration-time profile of idebenone in the mouse eye fluids. Following a single oral (gavage) administration of idebenone (60 mg/kg) to male mice, intra-ocular concentrations of idebenone reached a peak (C_max_) of 37.4 ng/ml (111nM) in the aqueous humor at the first sampling time 5 min post-dose (t_max_) (Fig. 3B and Tables S2, S3). The concentration-time profile of idebenone in the eye fluids was comparable to the plasma profile (Table S2), suggesting rapid uptake of idebenone into the plasma and the eye. Concentrations of idebenone in aqueous and vitreous humor generally declined over a period of 2 h post-dose (Fig. 3B and Table S2), suggesting an absence of sequestration of idebenone into the eye. The overall amount of idebenone in the aqueous humor, as measured by AUC_0-6h_, represented ∼10% of that observed in plasma (Table S3). Similarly, the concentration of idebenone in the vitreous humor represented ∼0.5% of that observed in plasma. These results indicate a rapid penetration of idebenone into the eye at concentrations shown to protect against rotenone-induced retinal ganglion cell death *in vitro*.

### Idebenone Protects Against Rotenone-induced RGC Death and Retinal Damage in vivo

Next, we investigated the efficacy of idebenone against consequences of complex I deficiency in the mouse retina *in vivo*. Mice were pre-treated for 3 weeks with idebenone or vehicle in the diet before rotenone was injected intravitrealy into the left eye. Retinal ganglion cell number, retinal thickness and reactive gliosis were quantitated based on immunohistochemical analysis of retinal sections 1 week after rotenone challenge (Fig. 4A–B). Animals pre-treated with dietary idebenone at 200, 400 and 2000 mg/kg/day were significantly protected against rotenone-induced RGC loss 7 days after the rotenone challenge when compared to vehicle-treated mice (Fig. 5A–B and Table 1). The injection-procedure itself caused no toxicity, as shown by comparable RGC numbers in sham-injected (63±9, n = 10) compared with non-injected eyes (50±6, n = 3). Consistent with this protection of RGCs, idebenone also prevented the decrease of retinal thickness 7 days after the rotenone challenge (Fig. 6A–B and Table 1). Similarly to RGCs, retinal thickness was not affected by the injection per se (sham-injected: 287±14 µm, n = 8; non-injected: 296±19 µm, n = 5). The extent of protection seen against rotenone-induced RGC loss and retinal thickness was comparable with the three doses of idebenone tested (Fig. 5B and 6B and Table 1). In addition, 7 days after rotenone injection, each of the idebenone doses tested significantly suppressed secondary pathological consequences of rotenone-mediated complex I inhibition, such as increased Müller cell gliosis (Fig. 7A–B and Table 1) and reduced glutamine synthetase expression (data not shown), which are markers of retinal pathology and cellular stress [32], [33]. These findings demonstrate that idebenone selectively protects against RGC loss, reduction of retinal thickness and reactive gliosis caused by complex I dysfunction in the retina.

### Idebenone Protects Against Rotenone-induced Loss of Vision in the Mouse in vivo

Since RGC loss is associated with visual acuity impairment [34] and idebenone protects against RGC loss and retinal damage, we investigated the functional effect of idebenone pre-treatment on visual acuity in animals with intravitreal injection of 5 mM rotenone using the optomotor head-tracking test (Fig. 8A, S1A and Video S1, S2). Intravitreal injection of 5 mM rotenone specifically abolished clockwise head responses entirely controlled by the left (injected) eye in all the treatment groups after 24 hours (Fig. 8B and Table S4). Consistent with the RGC protection previously observed, idebenone (2000 mg/kg) pre-treatment promoted a significant vision recovery 70 days after the rotenone injection when compared to vehicle-treated animals (Fig. 8B and Table S4). This protective effect of idebenone was also reflected by the increased number of mice displaying clockwise head responses (Fig. 8C). The effect of idebenone did not result from any change of vision related to the right (non-injected) eye since right eye-controlled counterclockwise head responses were similar in both idebenone- and vehicle-treated mice (Table S4). This is also evident when analyzing the ratio of clockwise and counterclockwise head responses (Fig. S1B). Moreover, sham-injected mice had no loss in clockwise head responses, suggesting no detrimental effect of the injection procedure itself (data not shown). These results demonstrate that idebenone preserves visual function in animals under conditions of impaired complex I function by protecting RGC and retinal integrity.

## Discussion

Leber’s hereditary optic neuropathy (LHON) is caused by mutations in complex I of the mitochondrial respiratory chain, which leads to complex I dysfunction, decreased ATP production and oxidative stress. The pathology of LHON is characterized by swelling of the retinal nerve fiber layer (RNFL) which precedes loss of retinal ganglion cells (RGC). This dynamic process evolves in parallel with functional impairment and atrophy of the nerve [6], [35]. Moreover, gliosis and decreased glutamine synthetase expression are consistent with optic nerve sheath distension [36], [37], [38] and glutamate transport impairment [14], [39], which can be observed in LHON patients. These pathologies, including the loss of optic nerve integrity, ultimately result in blindness of the patient. Here we demonstrated that idebenone is able to protect RGCs and retinal integrity against mitochondrial dysfunction *in vitro and in vivo.* Even though the mouse model used in this study might not replicate the condition in LHON patients in all aspects of the disease, these results strongly support the recently published data showing evidence for a therapeutic benefit of idebenone in prospective and retrospective clinical studies in LHON [40], [41], [42], and in a series of case reports where LHON patients favorably responded to idebenone [43], [44], [45], [46], [47], [48].

Since intra-ocular exposure with idebenone was not determined in the recent trials in LHON patients, it is of interest to compare the intra-ocular exposure that might result from dosing in humans at 900 mg idebenone/day (the dose used by [40] in the RHODOS trial) with the exposures found efficacious in the *in vitro* and *in vivo* models of LHON described here. This can be achieved by comparing the dose effective in the prevention of rotenone-induced cell death in the *in vivo* mouse model and in the clinical trial on a mg/kg and/or mg/m^2^ basis. The clinical dose of 900 mg/day idebenone (Catena®) was administered as 300 mg three times a day (tid) which corresponds to 15 mg/kg/day or 5 mg/kg tid for an average patient weight of 60 kg [40]. Taking a conservative approach, a single dose of 5 mg/kg in man (one third of the daily dose) represents a dose equivalent to 61.5 mg/kg in the mouse (using a generally accepted factor of 12.3 for an equivalent mg/kg dose for conversion from human to mouse; [49]). Assuming dose proportionality in plasma exposure, it can be expected that a dose of 61.5 mg/kg, given by gavage in the mouse would lead to an aqueous humor exposure of around 39 nM, well within the range shown to be effective *in vitro* (Fig. 1). This comparison therefore suggests that following a dose of 300 mg idebenone (Catena®) tid, the aqueous humor concentration of idebenone in man can be expected to reach levels in the same range as those shown to be effective in the *in vitro* and *in vivo* LHON animal models. Together with the LHON-like pathologies observed in the mouse model our results therefore not only support the clinical data but also validate the animal model used.

It is important to note that, in addition to its antioxidant function [22], [23], [25], [26], [50], idebenone can also maintain energy production under conditions of mitochondrial complex I deficiency by shuttling electrons directly from the cytoplasm to complex III [27], [28]. The process relies on NAD(P)H:quinone oxidoreductase 1 (NQO1), a cytosolic flavoprotein that catalyzes the reduction of idebenone [27], and is likely to contribute to the efficacy of idebenone in LHON. Thus, for the effectiveness of idebenone to restore energy production, expression of NQO1 or a similar reductase appears to be a fundamental requirement. Consistent with the protective effect of idebenone on RGCs shown in the LHON mouse model, NQO1 was found to be highly expressed in RGCs in the mouse retina (Fig. 2). The reason for the cell type-specific vulnerability of RGCs in LHON is still unclear but could lie at least in part in intrinsic features of RGCs which are known to have an extremely high energy demand [51] that makes them vulnerable to failure of mitochondrial energy production.

Based on our results, the potency of idebenone to alleviate symptoms and pathologies of LHON could thus be a consequence of its combined antioxidant function [22], [23], [24] and its ability to modulate mitochondrial electron flow [27], [28]. Since the visual system is the major energy consumer of the central nervous system, with a high susceptibility towards dysregulation of the energy metabolism [51], it is not surprising that a large number of ophthalmological disorders are associated with mitochondrial defects in energy metabolism and oxidative damage.

In summary, the data presented here, together with the favorable safety and tolerability profile of idebenone, provide an excellent rationale for the use of idebenone in LHON and open perspectives for future applications of idebenone in other ophthalmological indication.

## Supporting Information

Figure S1 **Idebenone protects against rotenone-induced loss of vision.** (a) Photograph of the experimental set up used for assessing visual acuity. (b) Visual acuity was also evaluated by counting the ratio between clock-wise and counterclock-wise head movements (CW/CCW) within 2 min. 7 days prior injection (day -7) and 1 to 70 days after injection (day 1, 7, 21, 35, 70) of 5 mM rotenone. Quantification following vehicle treatment and rotenone injection (vehicle + rotenone, n = 10 animals), and idebenone 2000 mg/kg treatment and rotenone injection (IDE 2000+ rotenone, n = 11 animals). Data are expressed as mean ± SEM.
(TIF)

Table S1 Concentrations of idebenone in plasma, aqueous and vitreous humor following repeated administration of idebenone in the diet to male mice. Data are expressed in ng/ml (± SEM). Idebenone was administered at 20, 40, 200, 400, and 2000 mg/kg in the diet. Idebenone concentration was measured at least 8 hours post last dose. blq: below limit of quantification (2 ng/ml); n = number of mice used for sampling; samples were pooled as outlined in Material and Methods section.
(DOCX)

Table S2 Pharmacokinetic profile of idebenone in plasma, aqueous and vitreous humor following single oral administration of idebenone at 60 mg/kg to male mice. Data are expressed in ng/ml (± SEM). Idebenone concentration was measured 5, 15, 30, 60, 120, 240, and 360 min post-dose. blq: below limit of quantification (2 ng/ml); n = number of mice used for sampling. Samples for aqueous and vitreous humor were pooled as outlined in the Material and Methods section.
(DOCX)

Table S3 Pharmacokinetic parameters of idebenone in plasma, aqueous and vitreous humor following single oral administration of idebenone at 60 mg/kg to male mice. Data are expressed as mean for plasma based on n = 20 mice per time point. Samples for aqueous and vitreous humor were pooled as outlined in the Material and Methods section.
(DOCX)

Table S4 Effect of idebenone (2000 mg/kg) treatment on head movements of mice with rotenone (5 mM)- and sham (DMSO)-injected eyes. Optomotor analysis of the rotenone (5 mM)-injected eye and the sham (DMSO)-injected eye in C57BL/6J mice was performed by counting clockwise (CW) and counterclockwise (CCW) head movements. Head movements were evaluated at a velocity of 3 rpm 7 days prior injection (day -7) and after injection (day 1, 7, 21, 35, 70). Optomotor data expressed as mean number of head movements/2 min. ± SEM. Statistical significance relative to vehicle + rotenone group: p≤0.05 (*) based on raw numbers; n = number of mice per group. CW: clockwise head movements; CCW: counterclockwise head movements; vehicle + rotenone: vehicle treated and injected with 5 mM rotenone; IDE2000+ rotenone: idebenone (2000 mg/kg) treated and injected with 5 mM rotenone.
(DOCX)

Video S1 **Normal clockwise (CW) and counter-clockwise (CCW) head movements during the optomotor response of a representative mouse, which was sham (DMSO)-injected into the eye.**
(WMV)

Video S2 **Absence of clockwise (CW) head movements during the optomotor response of a representative mouse, which was rotenone-injected into the eye.**
(WMV)
