# Supplementary figures and images for: Idebenone Protects against Retinal Damage and Loss of Vision in a Mouse Model of Leber’s Hereditary Optic Neuropathy
Source: PLoS One. 2012 Sep 18;7(9):e45182. doi: 10.1371/journal.pone.0045182 (PMC3445472; doi:10.1371/journal.pone.0045182)

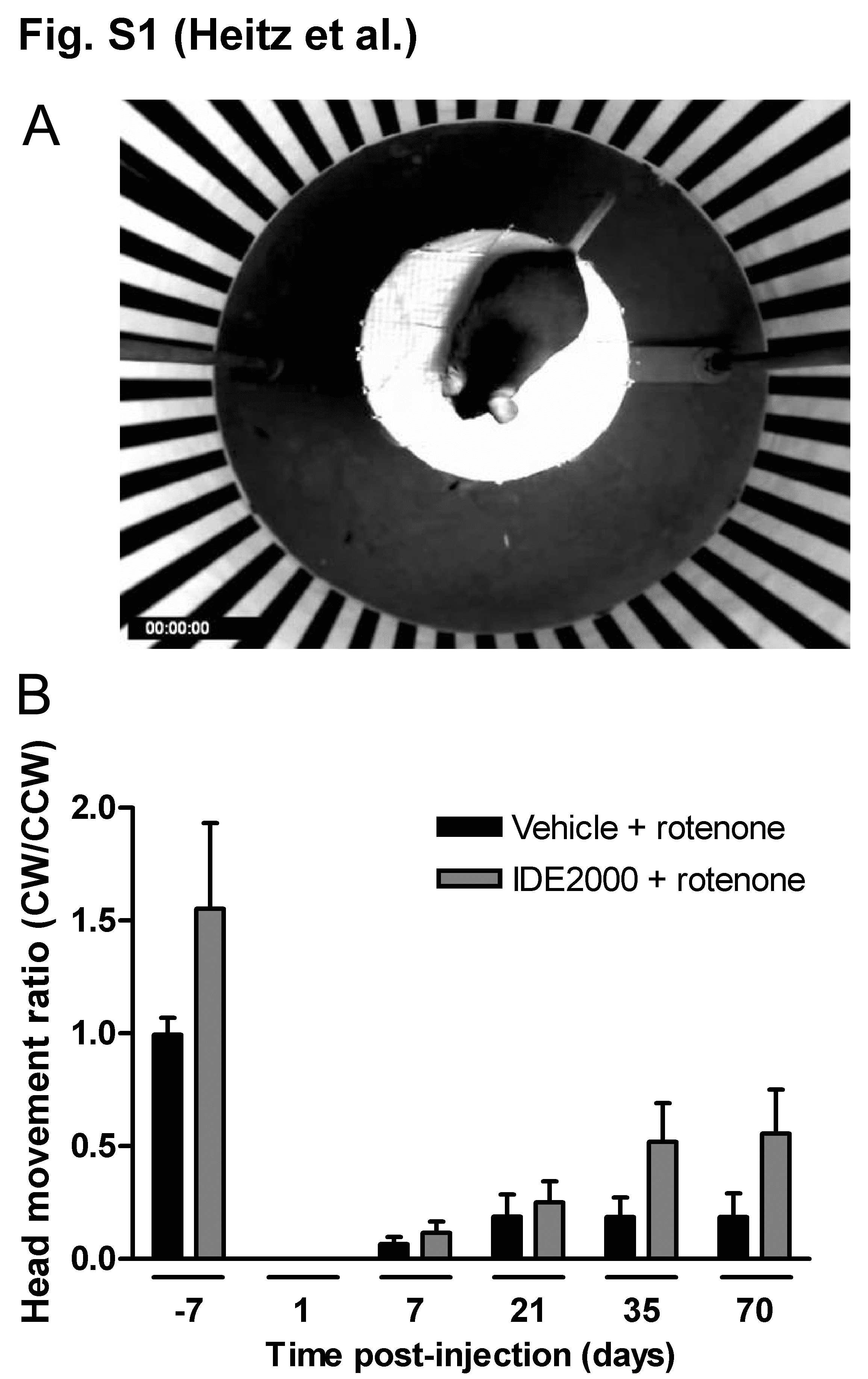

Supplement: Figure S1 — Idebenone protects against rotenone-induced loss of vision. (a) Photograph of the experimental set up used for assessing visual acuity. (b) Visual acuity was also evaluated by counting the ratio between clock-wise and counterclock-wise head movements (CW/CCW) within 2 min. 7 days prior injection (day -7) and 1 to 70 days after injection (day 1, 7, 21, 35, 70) of 5 mM rotenone. Quantification following vehicle treatment and rotenone injection (vehicle + rotenone, n = 10 animals), and idebenone 2000 mg/kg treatment and rotenone injection (IDE 2000+ rotenone, n = 11 animals). Data are expressed as mean ± SEM. (TIF) [file pone.0045182.s001.tif]
