# Supplementary material for: Idebenone Protects against Retinal Damage and Loss of Vision in a Mouse Model of Leber’s Hereditary Optic Neuropathy
Source: PLoS One. 2012 Sep 18;7(9):e45182. doi: 10.1371/journal.pone.0045182 (PMC3445472; doi:10.1371/journal.pone.0045182)
Supplement: Table S1 — Concentrations of idebenone in plasma, aqueous and vitreous humor following repeated administration of idebenone in the diet to male mice. Data are expressed in ng/ml (± SEM). Idebenone was administered at 20, 40, 200, 400, and 2000 mg/kg in the diet. Idebenone concentration was measured at least 8 hours post last dose. blq: below limit of quantification (2 ng/ml); n = number of mice used for sampling; samples were pooled as outlined in Material and Methods section. (DOCX) [file pone.0045182.s002.docx]

| Dose (mg/kg) | Idebenone (ng/ml) | | |
| --- | --- | --- | --- |
|  | Plasma | Aqueous | Vitreous |
| 0 | blq  (n=13) | blq  (n=20) | blq  (n=10) |
| 20 | blq  (n=15) | blq  (n=20) | blq  (n=20) |
| 40 | blq  (n=14) | blq  (n=20) | blq  (n=20) |
| 200 | 0.8 ± 0.4  (n=17) | 2.3  (n=20) | blq  (n=20) |
| 400 | 7.4 ± 3.3  (n=18) | 3.0  (n=20) | blq  (n=20) |
| 2000 | 44.7 ± 4.0  (n=19) | 12.6  (n=20) | 2.7 ± 0.1  (n=20) |
