# Supplementary material for: Idebenone Protects against Retinal Damage and Loss of Vision in a Mouse Model of Leber’s Hereditary Optic Neuropathy
Source: PLoS One. 2012 Sep 18;7(9):e45182. doi: 10.1371/journal.pone.0045182 (PMC3445472; doi:10.1371/journal.pone.0045182)
Supplement: Table S2 — Pharmacokinetic profile of idebenone in plasma, aqueous and vitreous humor following single oral administration of idebenone at 60 mg/kg to male mice. Data are expressed in ng/ml (± SEM). Idebenone concentration was measured 5, 15, 30, 60, 120, 240, and 360 min post-dose. blq: below limit of quantification (2 ng/ml); n = number of mice used for sampling. Samples for aqueous and vitreous humor were pooled as outlined in the Material and Methods section. (DOCX) [file pone.0045182.s003.docx]

| Time post-dose (min) | Idebenone (ng/ml) | | |
| --- | --- | --- | --- |
|  | Plasma | Aqueous | Vitreous |
| 5 | 474 ± 306 (n=20) | 37  (n=20) | 9 ± 5  (n=20) |
| 15 | 230 ± 45 (n=20) | 14  (n=20) | 2 ± 1  (n=20) |
| 30 | 241 ± 50 (n=20) | 16  (n=20) | blq  (n=20) |
| 60 | 60 ± 15 (n=20) | 14  (n=20) | blq  (n=20) |
| 120 | 7 ± 2  (n=20) | blq  (n=20) | blq  (n=20) |
| 240 | 3 ± 1  (n=20) | blq  (n=20) | blq  (n=20) |
| 360 | blq  (n=20) | blq  (n=20) | blq  (n=20) |
