# Supplementary material for: Idebenone Protects against Retinal Damage and Loss of Vision in a Mouse Model of Leber’s Hereditary Optic Neuropathy
Source: PLoS One. 2012 Sep 18;7(9):e45182. doi: 10.1371/journal.pone.0045182 (PMC3445472; doi:10.1371/journal.pone.0045182)
Supplement: Table S3 — Pharmacokinetic parameters of idebenone in plasma, aqueous and vitreous humor following single oral administration of idebenone at 60 mg/kg to male mice. Data are expressed as mean for plasma based on n = 20 mice per time point. Samples for aqueous and vitreous humor were pooled as outlined in the Material and Methods section. (DOCX) [file pone.0045182.s004.docx]

|  | Idebenone | | |
| --- | --- | --- | --- |
|  | Plasma | Aqueous | Vitreous |
| C_max_ (ng/ml) | 474 | 37.4 | 9.5 |
| C_max_ (nM) | 1402 | 111 | 28.1 |
| t_max_ (min) | 5 | 5 | 5 |
| AUC_0-6h_ (ng.h/ml) | 241 | 22.7 | 1.3 |
