# Supplementary material for: Idebenone Protects against Retinal Damage and Loss of Vision in a Mouse Model of Leber’s Hereditary Optic Neuropathy
Source: PLoS One. 2012 Sep 18;7(9):e45182. doi: 10.1371/journal.pone.0045182 (PMC3445472; doi:10.1371/journal.pone.0045182)
Supplement: Table S4 — Effect of idebenone (2000 mg/kg) treatment on head movements of mice with rotenone (5 mM)- and sham (DMSO)-injected eyes. Optomotor analysis of the rotenone (5 mM)-injected eye and the sham (DMSO)-injected eye in C57BL/6J mice was performed by counting clockwise (CW) and counterclockwise (CCW) head movements. Head movements were evaluated at a velocity of 3 rpm 7 days prior injection (day -7) and after injection (day 1, 7, 21, 35, 70). Optomotor data expressed as mean number of head movements/2 min. ± SEM. Statistical significance relative to vehicle + rotenone group: p≤0.05 (*) based on raw numbers; n = number of mice per group. CW: clockwise head movements; CCW: counterclockwise head movements; vehicle + rotenone: vehicle treated and injected with 5 mM rotenone; IDE2000+ rotenone: idebenone (2000 mg/kg) treated and injected with 5 mM rotenone. (DOCX) [file pone.0045182.s005.docx]

| Treatment group | Day -7 | | Day 1 | | Day 7 | |
| --- | --- | --- | --- | --- | --- | --- |
|  | CW | CCW | CW | CCW | CW | CCW |
| Vehicle + rotenone (n=10) | 8.4 ± 0.6 | 8.4 ± 0.3 | 0 ± 0 | 12.6 ± 0.4 | 0.8 ± 0.3 | 12.2 ± 0.4 |
| IDE2000 + rotenone (n=11) | 8.8 ± 0.7 | 7.4 ± 0.8 | 0 ± 0 | 12.7 ± 0.4 | 1.5 ± 0.6 | 12.3 ± 0.5 |

| Treatment group | Day 21 | | Day 35 | | Day 70 | |
| --- | --- | --- | --- | --- | --- | --- |
|  | CW | CCW | CW | CCW | CW | CCW |
| Vehicle + rotenone (n=10) | 2.8 ± 1.6 | 14.3 ± 0.9 | 2.3 ± 1.0 | 13.3 ± 0.7 | 2.1 ± 1.1 | 13.6 ± 1.3 |
| IDE2000 + rotenone (n=11) | 3.2 ± 1.2 | 12.9 ± 0.8 | 4.0 ± 1.1 | 9.9 ± 0.8* | 5.0 ± 1.2* | 12.1 ± 1.2 |
